# Racial Disparities In In-Hospital Mortality of Children and Adolescents Under 20 Years With Type 1 Diabetes Mellitus

**DOI:** 10.7759/cureus.43999

**Published:** 2023-08-23

**Authors:** Nnamdi J Omenuko, Yordanos Tafesse, Hezborn M Magacha, Valentine C Nriagu, Sandra O Anazor, Chisom M Nwaneki, Francis Okeke, Chimezirim Ezeano, Chukwuma Jideofor

**Affiliations:** 1 Hematology and Oncology, The University of Chicago Medicine, Chicago, USA; 2 Internal Medicine, East Tennessee State University, Johnson City, USA; 3 Epidemiology and Public Health, East Tennessee State University, Johnson City, USA; 4 Internal Medicine, Maimonides Medical Center, New York, USA; 5 Obstetrics and Gynecology, Corewell Health West/Michigan State University, Grand Rapids, USA; 6 Public Health, East Tennessee State University, Johnson City, USA; 7 Internal Medicine, Saint Peter’s University Hospital, New Brunswick, USA; 8 Epidemiology and Public Health, University of North Texas Health Science Center, Fort Worth, USA; 9 Ophthalmology, National Hospital Abuja, Abuja, NGA

**Keywords:** united states of america, adolescent diabetes, child and adolescent, racial disparity, type i diabetes mellitus

## Abstract

Background: In the United States, racial disparities in health outcomes continue to be a major problem with far-reaching effects on equity in healthcare and public health. Children and teenagers with type 1 diabetes are a disadvantaged demographic that has particular difficulties in managing their condition and getting access to healthcare. Despite improvements in the treatment of diabetes, little study has examined how much racial disparities in in-hospital mortality affect this particular demographic. By examining racial differences in in-hospital mortality rates among children and adolescents with type 1 diabetes in the United States, this study seeks to close this gap.

Methods: This cross-sectional study utilized data from the Healthcare Cost and Utilization Project's (HCUP) Kids' Inpatient Database (KID) for 2012. The KID is a nationally representative sample of pediatric discharges from US hospitals. A total of 20,107 patients who were admitted with type 1 diabetes were included in this study. The primary outcome was the patient's in-hospital mortality status. The primary predictor variable was the race of the patient. Six potential confounders were chosen based on previous literature: age, sex, hospital location, obesity, weight loss, electrolyte disorders status, and median household income. Descriptive statistics and bivariate analyses were done. Multivariate analysis was conducted while controlling for potential confounders. Odd ratios with a 95% confidence interval and probability value were reported. Statistical Analysis System (SAS) version 9.4 for Windows (SAS Institute Inc., Cary, NC, USA) was used for the statistical analysis.

Results: A total of 20,107 patients were included in this study. Of the patients included, 78.6%, 5.3%, 5.9%, and 10.2% were of age groups <4, 5-9, 10-14, and 15-18, respectively. Among the patients, 64.3% were female. Whites stood at 54.3%, while Hispanic, Black, and other races accounted for 17.2%, 21.8%, and 6.7% respectively. After adjusting for all other variables, children, and young adults of Asian and Pacific Islanders (OR=1.948; 95% CI 1.015,3.738) had 94% higher odds of in-hospital mortality compared to their White counterparts. Children and young adults aged 5-9 (OR=0.29; 95% CI 0.13,0.649) had 71% lower odds of in-hospital mortality compared to those aged 4 or under. Those aged 10-14 (OR=0.155; 95% CI 0.077,0.313) had 85% lower odds of in-hospital mortality compared to those aged 4 or under, while those aged 15-19 (OR=0.172; 95% CI 0.100,0.296) had 83% lower odds of in-hospital mortality compared to those aged 4 or under. Children and young adults who had weight loss (OR=4.474; 95% CI 2.557,7.826) had almost five times higher odds of in-hospital mortality compared to those without weight loss, while children and young adults who had electrolyte disorders (OR=5.131; 95% CI 3.429,7.679) had five times higher odds of in-hospital mortality compared to those without electrolyte disorders.

Conclusion: The results show young adults of Asian and Pacific Islanders have higher odds of in-hospital mortality compared to their White counterparts and this study highlights the urgent need for focused measures designed to lessen these inequalities and enhance health equity. The implementation of culturally sensitive healthcare practices, addressing social determinants of health, and enhancing access to high-quality diabetes care should all be priorities.

## Introduction

Type 1 diabetes is a common chronic disease of childhood affecting approximately 200,000 children and adolescents in the United States [1-4]. Children and adolescents with diabetes are at an increased risk of death from acute complications of diabetes, including hypoglycemia and diabetic ketoacidosis. Type I diabetes can lead to heart disease, kidney disease, and other cerebrovascular events which can lead to serious complications and even death [1-3]. According to the Centers for Disease Control and Prevention (CDC), 1.6 million people have type 1 diabetes and the incidence of type 1 diabetes is increasing, with an estimated 40,000 new cases diagnosed each year in the United States [1,2].

Despite the higher prevalence and incidence of reported diabetes among Whites than Blacks, the CDC however, noted disparities by skin color during 1979-2004, with Black children and adolescents dying from diabetes at double the rate of White children and adolescents, and thrice the rate of their Hispanic counterparts. The CDC performed analysis using data from the National Vital Statistics System for deaths among persons aged 1-19 years in the United States during 2000-2014, with diabetes listed as the predisposing cause of death overall. During 2012-2014, Black children and young adults had the highest diabetes-related death rates (2.04 per 1 million population), followed by Whites (0.92) and Hispanics (0.61) [1,3,4]. Over the study period, there were no statistically significant changes in diabetes death, but differences persisted among racial groups. Diabetes-related deaths in children and adolescents are potentially preventable through increased awareness of diabetes symptoms (including symptoms of decreased blood glucose), earlier treatment, education related to diabetes, and management of diabetic ketoacidosis. Continued measures are needed to lower diabetes deaths in children and understand the reasons for racial and ethnic disparities.

Young adults of racial/ethnic minorities are the largest growing populations with type 1 diabetes and exhibit very negative outcomes, making it important to understand the specific needs and challenges of these groups [3,5-7]. The reasons for these differences are complex and involve many factors, including a combination of biological, social, and economic factors. Biological factors that contribute to the increased risk of diabetes in racial minorities include a higher prevalence of obesity, insulin resistance, and genetic predisposition. Social factors such as limited access to healthy food options and safe environments for physical activity, also play a significant role. Additionally, economic factors, such as poverty and lack of access to quality healthcare, can also contribute to the higher prevalence of diabetes and diabetes-related complications in these populations [5,8,9]. Diabetes technology therapies are proliferating and offer better options for attaining glycemic control and preventing long-term complications. However, recent studies have shown that minority populations, especially young adults, are among the lowest consumers of these recent technologies, which may contribute to observed disparities in glycemic controls [10-13].

Equally important to consider is the role of healthcare providers in creating disparities. Several studies have demonstrated that healthcare racism and implicit bias exist, even in well-meaning engaged providers [2,14,15]. The study aims to identify whether there are significant differences in in-hospital mortality rates between racial and ethnic groups and to explore potential factors that contribute to these disparities. The goal of this study is to inform public health policies and interventions aimed at reducing racial disparities in diabetes care and improving health outcomes for all children and young adults with type 1 diabetes.

## Materials and methods

This cross-sectional study utilized data from the Healthcare Cost and Utilization Project's (HCUP) Kids' Inpatient Database (KID) for 2012. The KID is a nationally representative sample of pediatric discharges from US hospitals. A total of 20,107 patients who were admitted with type 1 diabetes were included in this study. The primary outcome was the patient's in-hospital mortality, which represented if the patient died during hospitalization. A new variable "DMortality" was created to describe the patients who had diabetes and died during hospitalization. This new variable was recorded into a binary categorical variable. Our primary predictor variable was the race of the patient, it was recorded into 4 strata variables which are White, Black, Hispanic, and "other races." Asian, Pacific Islander, Native American, and other races were recorded as "other races." Six potential confounders were chosen based on previous literature: age, sex, hospital location, obesity, weight loss, electrolyte disorders status, and median household income. Age, originally a continuous variable, was recoded into a 4-level categorical variable: <4 years, 5-9 years, 10-14 years, and 15-18 years. The data were weighted using the sampling weight.

Descriptive statistics were done for all variables. The percentage of patients who had type 1 diabetes and died during hospitalization was calculated and it was assessed by the patient’s age, sex, hospital location, obesity status, weight loss status, electrolyte disorders status, and median household income. The chi-square test was used to test the significance of the differences in proportion between groups. Bivariate analyses were done using simple logistic regression to assess the relationship between each of our predictor variables (age, sex, hospital location, obesity, weight loss, electrolyte disorders status, and median household income) and the outcome variable (in-hospital mortality). Multivariate analysis was conducted using multivariate logistic regression models to assess the association between the primary predictor variable (race) and the outcome variable (in-hospital mortality) while controlling for potential confounders (age, sex, hospital location, obesity, weight loss, electrolyte disorders status, and median household income). Odd ratios with a 95% confidence interval and probability value were reported for both the bivariate and multivariate analyses. Statistical software Statistical Analysis System (SAS) version 9.4 for Windows (SAS Institute Inc., Cary, NC, USA) was used for the statistical analysis.

## Results

Descriptive analysis

A total of 20,107 patients were included in this study. Of the patients included, 78.6%, 5.3%, 5.9%, and 10.2% were of age groups <4, 5-9, 10-14, and 15-18, respectively (Table 1). Among the patients, 64.3% were female. Whites stood at 54.3%, while Hispanics, Blacks and other races accounted for 17.2%, 21.8%, and 6.7% respectively (Table 1). Regarding hospital location, rural areas, urban non-teaching, and urban teaching areas managed 2.5%, 15.4%, and 82.1% of the total number of patients respectively. Among the patients, 13.8% were obese, 17.9% of patients had weight loss, and 56% had electrolyte disorders. All relationships were significant in the chi-square analysis at p<0.05 level.

**Table 1:** Frequency distribution of baseline patient characteristics by in-hospital mortality (weighted)

	No in-hospital mortality N=19976	In-hospital mortality N=131	
Variable	N (%)	N (%)	p-value
Age			<0.0001
<4	841 (5.02%)	22 (16.7%)	
5-9	1003 (6.12%)	13 (9.92%)	
10-14	3451 (21.08%)	16 (12.2%)	
15-19	11081 (67.103%)	60 (45.8%)	
Sex			0.0005
Female	12955 (64.04%)	65 (49.7%)	
Male	7083 (35.31%)	66 (50.2%)	
Race			0.1101
Black	4042 (21.7%)	22 (18.5%)	
Hispanic	3221 (17.06%)	20 (17.0%)	
White	10051 (53.26%)	63 (52.3%)	
Other	1258 (6.65%)	15 (12.2%)	
Location of hospital (recoded from)			0.0185
Urban non-teaching	4294 (20.7%)	21 (15.4%)	
Urban teaching	14324 (71.1%)	107 (82.1%)	
Rural	1358 (7.4%)	3 (2.5%)	
Obesity status			0.8394
No	17084 (85.01%)	113 (86.2%)	
Yes	2892 (14.34%)	18 (13.8%)	
Weight loss			<0.0001
No	19014 (94.46%)	107 (82.1%)	
Yes	962 (4.89%)	24 (17.9%)	
Electrolyte disorder			<0.001
No	16320 (81.12%)	57 (43.6%)	
Yes	3656 (18%)	74 (56.4%)	

Bivariate and multivariate analysis 

Bivariate analysis revealed Asian and Pacific Islanders (OR=1.903, 95% CI 1.080,3.351), weight loss (OR=4.435, 95% CI 2.835,6.937), electrolyte disorder (OR=5.795, 95% CI 4.095,8.201), urban teaching hospital (OR=3.381, 95% CI 1.072,10.665), and males (OR=1.848, 95% CI 1.310,2.606) were also associated with higher odds of in-hospital mortality compared to their counterparts (Table 2). Children and young adults aged 5-9 (OR=0.495; 95% CI 0.248,0.989), those aged 10-14 (OR=0.177, 95% CI 0.093,0.339), and those aged 15-19 (OR=0.207, CI 0.216,0.339) were associated with lower odds of in-hospital mortality compared to those aged <4 years of age.

**Table 2:** Adjusted and unadjusted analysis of race and in-hospital mortality in type 1 diabetes

	Unadjusted		Adjusted	
Variable		p-value		p-value
Race				
White	Ref	Ref	Ref	Ref
Black	0.868	0.5698	0.965	0.9041
Hispanic	0.991	0.9708	1.195	0.5301
Other	1.903	0.0259	1.948	0.0463
Age				
<4	Ref	Ref	Ref	Ref
5-9	0.495	<0.0001	0.29	0.0465
10-14	0.177	<0.0001	0.155	<0.0001
15-19	0.207	<0.0001	0.172	<0.0001
Sex				
Female	Ref	Ref	Ref	Ref
Male	1.848	0.0005	1.383	0.1155
Location of hospital				
Rural	Ref	Ref	Ref	Ref
Urban non-teaching	2.214	0.1985	1.629	0.4507
Urban teaching	3.381	0.0376	2.361	0.1552
Obesity status				
No	Ref	Ref	Ref	Ref
Yes	0.941	0.8112	1.118	0.7369
Weight loss				
No	Ref	Ref	Ref	Ref
Yes	4.435	<0.0001	4.474	<0.0001
Electrolyte disorders				
No	Ref	Ref	Ref	Ref
Yes	5.795	<0.0001	5.131	<0.0001

After adjusting for all other variables, children, and young adults of Asian and Pacific Islanders (OR=1.948; 95% CI 1.015,3.738) had 94% higher odds of in-hospital mortality compared to their White counterparts (Table 2). Children and young adults aged 5-9 (OR=0.29; 95% CI 0.13,0.649) had 71% lower odds of in-hospital mortality compared to those aged 4 or under. Those aged 10-14 (OR=0.155; 95% CI 0.077,0.313) had 85% lower odds of in-hospital mortality compared to those aged 4 or under, while those aged 15-19 (OR=0.172; 95% CI 0.100,0.296) had 83% lower odds of in-hospital mortality compared to those aged 4 or under (Table 2). Children and young adults who had weight loss (OR=4.474; 95% CI 2.557,7.826) had almost five times higher odds of in-hospital mortality compared to those without weight loss, while children and young adults who had electrolyte disorders (OR=5.131; 95% CI 3.429,7.679) had five times higher odds of in-hospital mortality compared to those without electrolyte disorders. 

## Discussion

In this study, we examined type 1 diabetes patients who have been hospitalized. We wanted to determine the racial disparities in in-hospital mortality in this category of patients and explore other differences reported by age, sex, median household income, hospital location, obesity, and electrolyte disorders. Our results indicated that there were racial disparities in in-hospital mortality with children and young adults of Asian and Pacific Islanders having higher odds of in-hospital mortality compared to their White counterparts [3,7,8,16]. This is consistent with similar studies that showed that young adults of racial/ethnic minorities exhibit very negative outcomes, making it important to understand the specific needs and challenges of these groups. 

The causes of these variations are multifaceted and may include a variety of biological, social, and economic factors. Higher rates of obesity, insulin resistance, and genetic predisposition are a few biological reasons that raise the risk of diabetes in ethnic minorities. Social issues, such as the lack of access to healthy food options and unsafe venues for exercise, are also quite important. Technology-based treatments for diabetes are becoming more common and provide better choices for achieving glycemic control and avoiding long-term problems. However, recent research has revealed that minority communities, particularly young individuals, are among the least likely to use these contemporary technologies, which could explain observed differences in glycemic control [4,10,14,16].

Through better knowledge of diabetes symptoms (including signs of low blood sugar), early diagnosis, diabetes education, and proper management of diabetic ketoacidosis, diabetes-related mortality in children and adolescents may be avoided. To reduce childhood diabetes fatalities and comprehend the causes of racial and ethnic differences, ongoing efforts are required. According to the study's findings, there are racial differences in the in-hospital death rates for children and young people with type 1 diabetes mellitus. Since diabetes is a serious and expanding issue in the United States, particularly among children and young adults, these differences have substantial public health consequences. To address this issue, it is important to consider both healthcare policy changes and individual interventions that can help to reduce disparities and improve outcomes for all patients.

Increasing access to healthcare services and resources in underserved communities is one potential policy adjustment that could aid in reducing racial disparities in in-hospital mortality for this population. This can entail sponsoring community health centers and contributing to initiatives for diabetes education and prevention in community centers and schools. Patients from underserved communities may be more likely to obtain appropriate and timely care if they have more access to healthcare services and resources, which could lower the risk of complications and improve overall health outcomes. Additionally, by providing culturally competent treatment and addressing unintentional biases and prejudices, healthcare providers can significantly contribute to the reduction of disparities. This could include providing interpreter services for non-English speaking patients, using patient-centered communication strategies, and working with community organizations to promote diabetes education and prevention. By working together to address this important public health issue, we can help to reduce morbidity, mortality, and healthcare costs associated with diabetes, and improve outcomes for patients of all races and backgrounds.

Limitations 

The HCUP KID dataset is an administrative database, and as such, may be subject to coding errors, inaccuracies, and missing data. The study was limited to in-hospital mortality, and therefore, may not capture deaths that occur outside of the hospital, such as at home or in hospice care. The study focused on children and young adults with type 1 diabetes and may not be generalizable to other age groups or individuals with type 2 diabetes mellitus. Additionally, the findings may not be generalizable to other geographic regions or healthcare settings.

## Conclusions

The results of this study may have significant effects on public health and provide guidance for programs and policies aimed at minimizing racial disparities in health outcomes. The study's findings may be used to educate politicians and healthcare professionals about the importance of paying more attention to racial inequities in diabetes care. The report specifically contends that there may be racial disparities in healthcare outcomes and that every child and adolescent with type 1 diabetes deserves access to high-quality care, regardless of their race or ethnicity. Healthcare professionals can promote routine diabetes screenings for all children and young adults, especially those from racial and ethnic minority groups, expand access to diabetes medications and supplies, and establish culturally sensitive diabetes education programs. Policies that increase financing for diabetes preventive and management programs and broaden insurance coverage for diabetic care are just two examples of how policymakers might improve access to high-quality diabetes care. Raising awareness of racial inequities in diabetes care and promoting legislation to remedy them are tasks for advocacy groups. Stakeholders can enhance diabetes treatment and lessen racial disparities in health outcomes by cooperating and using the study's findings.
